# Molecular mechanisms underlying therapeutic potential of pericytes

**DOI:** 10.1186/s12929-018-0423-7

**Published:** 2018-03-09

**Authors:** C. Randall Harrell, Bojana Simovic Markovic, Crissy Fellabaum, Aleksandar Arsenijevic, Valentin Djonov, Vladislav Volarevic

**Affiliations:** 1 Regenerative Processing Plant, LLC, 34176 US Highway 19 N Palm Harbor, Palm Harbor, Florida USA; 2 Department of Microbiology and immunology, Center for Molecular Medicine and Stem Cell Research, University of Kragujevac, Serbia, Faculty of Medical Sciences, 69 Svetozar Markovic Street, Kragujevac, 34000 Serbia; 3 University of Bern, Institute of Anatomy, Baltzerstrasse 2, Bern, Switzerland

**Keywords:** Pericytes, Cell therapy, Vascular disorders

## Abstract

**Background:**

Pericytes are multipotent cells present in every vascularized tissue in the body. Despite the fact that they are well-known for more than a century, pericytes are still representing cells with intriguing properties. This is mainly because of their heterogeneity in terms of definition, tissue distribution, origin, phenotype and multi-functional properties. The body of knowledge illustrates importance of pericytes in the regulation of homeostatic and healing processes in the body.

**Main body:**

In this review, we summarized current knowledge regarding identification, isolation, ontogeny and functional characteristics of pericytes and described molecular mechanisms involved in the crosstalk between pericytes and endothelial or immune cells. We highlighted the role of pericytes in the pathogenesis of fibrosis, diabetes-related complications (retinopathy, nephropathy, neuropathy and erectile dysfunction), ischemic organ failure, pulmonary hypertension, Alzheimer disease, tumor growth and metastasis with the focus on their therapeutic potential in the regenerative medicine. The functions and capabilities of pericytes are impressive and, as yet, incompletely understood. Molecular mechanisms responsible for pericyte-mediated regulation of vascular stability, angiogenesis and blood flow are well described while their regenerative and immunomodulatory characteristics are still not completely revealed. Strong evidence for pericytes’ participation in physiological, as well as in pathological conditions reveals a broad potential for their therapeutic use. Recently published results obtained in animal studies showed that transplantation of pericytes could positively influence the healing of bone, muscle and skin and could support revascularization. However, the differences in their phenotype and function as well as the lack of standardized procedure for their isolation and characterization limit their use in clinical trials.

**Conclusion:**

Critical to further progress in clinical application of pericytes will be identification of tissue specific pericyte phenotype and function, validation and standardization of the procedure for their isolation that will enable establishment of precise clinical settings in which pericyte-based therapy will be efficiently applied.

## Background

Pericytes are multipotent cells present in every vascularized tissue in the body [1]. They were first described in the 1871 by a German pathologist and bacteriologist Carl Joseph Eberth and “rediscovered” 2 years later by French physiologist and anatomist Charles-Marie Benjamin Rouget who defined them as a mural cell population embedded in the basement membrane of venules and capillaries [2]. The “godfather” of pericytes was German anatomist and histologist Karl Wilhelm Zimmermann who gave them their current name in 1923, by using the Greek term “*kytos*”-meaning a hollow vessel, to appropriately describe a cell surrounding a blood vessel [3]. Despite the fact that they have been well-known for more than a century, pericytes are still representing cells with intriguing properties. This is mainly because of their heterogeneity in terms of definition, tissue distribution, embryonic origin, phenotype and multi-functional properties [1].

Currently, a mature pericyte is defined as a “cell embedded within the vascular basement membrane” [4]. Another commonly applied defining criterion is the presence in microvessels, (capillaries, postcapillary venules, and terminal arterioles), but this definition has been challenged by observations of subendothelial pericyte-like cells in large vessels [5]. Pericytes, vascular smooth muscle cells (vSMCs), fibroblasts, macrophages, and even epithelial cells can be found in the periendothelial location. Since there is no available molecular marker that can be used to specifically distinguish pericytes from vSMCs or other mesenchymal cells, the term pericyte is often used in the literature to designate any microvascular periendothelial mesenchymal cell. Thus, as a compromise nowadays, pericytes are usually defined by using several criteria including location, morphology, and expression of several markers.

Herewith, we summarized current knowledge regarding identification, isolation, ontogeny and functional characteristics of pericytes and described molecular mechanisms involved in crosstalk between pericytes and endothelial cells (ECs) or immune cells. Additionally, we highlighted the role of pericytes in the pathogenesis of several diseases with the focus on their therapeutic potential in the regenerative medicine.

## Origin, isolation, identification and morphology of pericytes

Pericytes are heterogenous in terms of embryonic origin. Ectoderm-derived neural crest gives rise to pericytes in the central nervous system and thymus while in lung, heart, liver and gut, the mesothelium is the main source of pericytes. In most other organs, pericytes derive from the mesoderm; specifically, the sclerotomal compartment [6]. Interestingly, it was recently revealed that some of the pericytes in the embryonic skin and brain had hematopoietic origin indicating that pericytes within the same tissue may be heterogeneous in their origin [7].

For research purposes, pericytes were most usually isolated from bovine retina or brain since the highest density of pericytes to ECs has been observed in these organs [8]. The other alternative sources for pericytes isolation were skeletal muscle, adipose tissue, skin, placenta, umbilical cord, bone marrow, kidney and liver [9, 10].

**Fig. 1:**
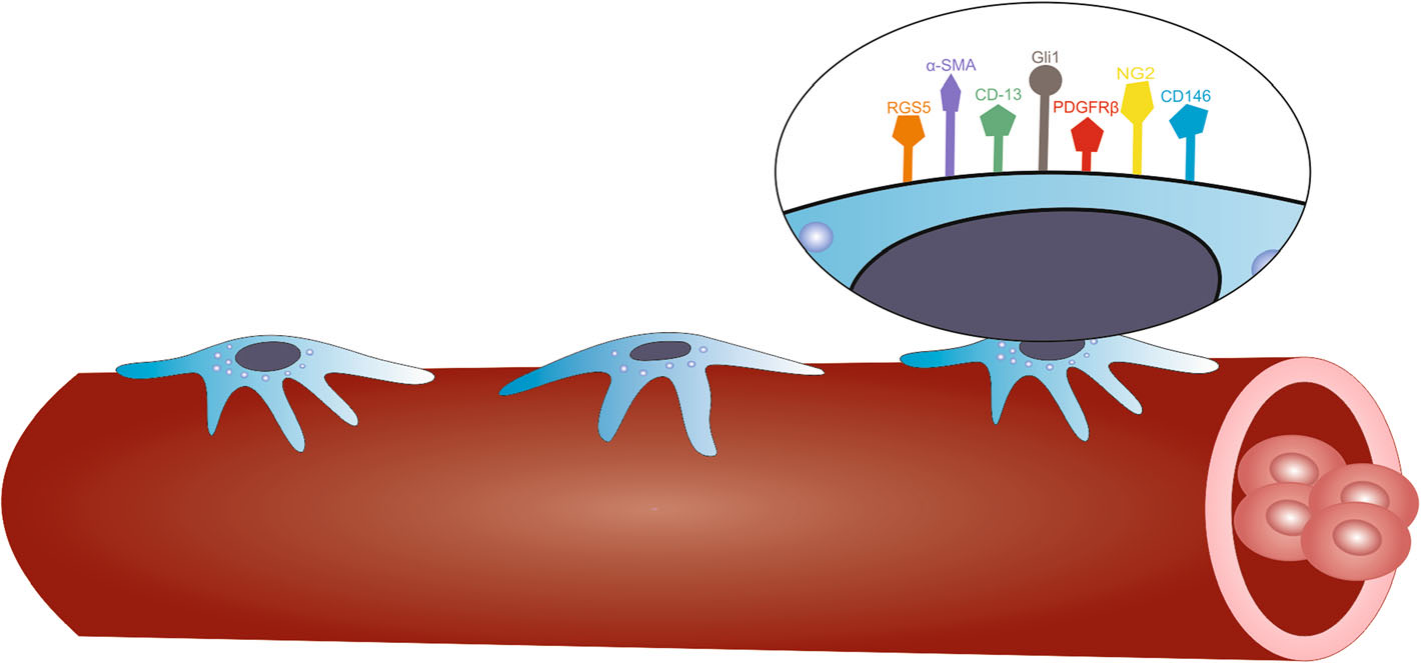
Markers of pericytes identification. Due to the heterogeneity of pericytes, several markers were usually used for their identification such as platelet-derived growth factor receptor β (PDGFRβ): receptor with tyrosin kinase activity, involved in pericytes proliferation and recruitment; nerve-glial antigen-2 (NG2): membrane chondroitin sulfate proteoglycan involved in pericyte recruitment to tumor vasculature; CD146: transmembrane glycoprotein that functions as a Ca2 + −independent cell adhesion molecule; the regulator of G-protein signaling-5 (RGS5): a GTPase-activating protein, expressed on activated pericytes during vessel remodeling and tumor development; α-smooth muscle actin (α-SMA) and desmin: structural proteins, important for pericyte contraction and regulation of blood pressure; aminopeptidase N (CD13): membrane zinc-dependent metalloprotease, expressed mainly on brain pericytes; glioma-associated oncogene (Gli1): zinc finger protein, effector of Hedgehog signaling pathway, involved in pericyte-mediated modulation of fibrosis and in the maintenance of peritubular capillary health and T-box transcription factor TBX18 (Tbx18): involved in the development of the heart and coronary vessels

Due to the heterogeneity of pericytes, several markers were usually used for their identification (Fig. 1): platelet-derived growth factor receptor β (PDGFRβ): receptor with tyrosin kinase activity, involved in pericytes proliferation and recruitment [11]; nerve-glial antigen-2 (NG2): membrane chondroitin sulfate proteoglycan involved in pericyte recruitment to tumor vasculature [12]; CD146: transmembrane glycoprotein that functions as a Ca2 + −independent cell adhesion molecule [13]; the regulator of G-protein signaling-5 (RGS5): a GTPase-activating protein, expressed on activated pericytes during vessel remodeling and tumor development [14]; α-smooth muscle actin (α-SMA) and desmin: structural proteins, important for pericyte contraction and regulation of blood pressure; aminopeptidase N (CD13): membrane zinc-dependent metalloprotease, expressed mainly on brain pericytes; glioma-associated oncogene (Gli1): zinc finger protein, effector of Hedgehog signaling pathway, involved in pericyte-mediated modulation of fibrosis and in the maintenance of peritubular capillary health [15, 16] and T-box transcription factor TBX18 (Tbx18): involved in the development of the heart and coronary vessels [17]. Importantly, none of these markers are pericyte specific; since they are also expressed by other cell types [for example PDGFRβ is a well-known marker of fibroblasts [18], while NG2 is expressed on macrophages] [5]. Moreover, not all pericytes express every single marker; their expression is dynamic and varies between organs (Table 1), developmental stages, activation/maturation state, and across individual microvascular networks.

**Table 1:** The differences of pericyte markers in different tissues and organs

Tissue/organ	Markers
Brain	• potassium channel complex Kir6.1+ • Nerve-glial antigen-2 (NG2)+ • Desmin+ • Platelet-derived growth factor receptor β (PDGFRβ)+ [19]
Retina	• Nerve-glial antigen-2 (NG2)+ • Platelet-derived growth factor receptor β (PDGFRβ)+ • α-smooth muscle actin (α-SMA)- [20]
Kidney	• Endosialin (CD248)+ • Platelet-derived growth factor receptor β (PDGFRβ)+ • ecto-5′-nucleotidase (CD73)+ • collagen (Col)1a1+ [21]
Skeletal muscle	• alkaline phosphatase+ • Nerve-glial antigen-2 (NG2)+ • α-smooth muscle actin (α-SMA)+ • Desmin+ [22]

Morphology of pericytes is determined by their localization and the content of α-SMA. Mid-capillary pericytes have lack of α-SMA and are elongated and more spindle shaped, while pre- and post-capillary pericytes contain varying amounts of α-SMA and are shorter and stellate-shaped [23]. The typical mature capillary pericyte has a nearly rounded cell body characterized by minimal cytoplasm, a prominent, discoid nucleus and projecting processes which wrap around associated capillaries [24]. The primary processes give rise to secondary perpendicular processes which are firmly attached to the endothelium by their tips [5]. Microtubules stretch along the primary and secondary cytoplasmic extensions. Desmin and vimentin-containing intermediate filaments are mostly concentrated within the primary extensions while actin, myosin, and tropomyosin-containing microfilaments are concentrated beneath the plasma membrane, in inner surface membrane facing the endothelium. The outer, abluminal pericyte surface is characterized by the presence of numerous flask-shaped or spherical invaginations of the plasma membrane, named plasmalemmal vesicles or caveolae [5].

From their position on the outer surface of the blood vessel, pericytes interact with ECs, which reside on the other side of the basement membrane. The number and size of pericyte-endothelial contacts vary between tissues, and can increase up to 1000 for a single endothelial cell. The majority of these contacts are of peg-socket type, in which pericyte cytoplasmic fingers (pegs) are inserted into endothelial invaginations (pockets). Other contact morphologies include occluding contacts and adhesion plaques that strengthen adherence between ECs and pericytes. The occluding contacts are located at the edge of the pericyte processes and through them membranes of pericytes and ECs come very close to each other enabling straightway contact between these cells. The adhesion plaques, which provide adherence between the ECs and the pericytes, represent fibronectin-reach microfilament bundles attached at the pericyte plasma membrane and electron-dense material in the opposing endothelial cytoplasm. These are the sites where N-cadherin-based connections are formed between ECs and pericytes [5, 14, 25].

## Signaling pathways involved in the crosstalk between pericytes and ECs

**Fig. 2:**
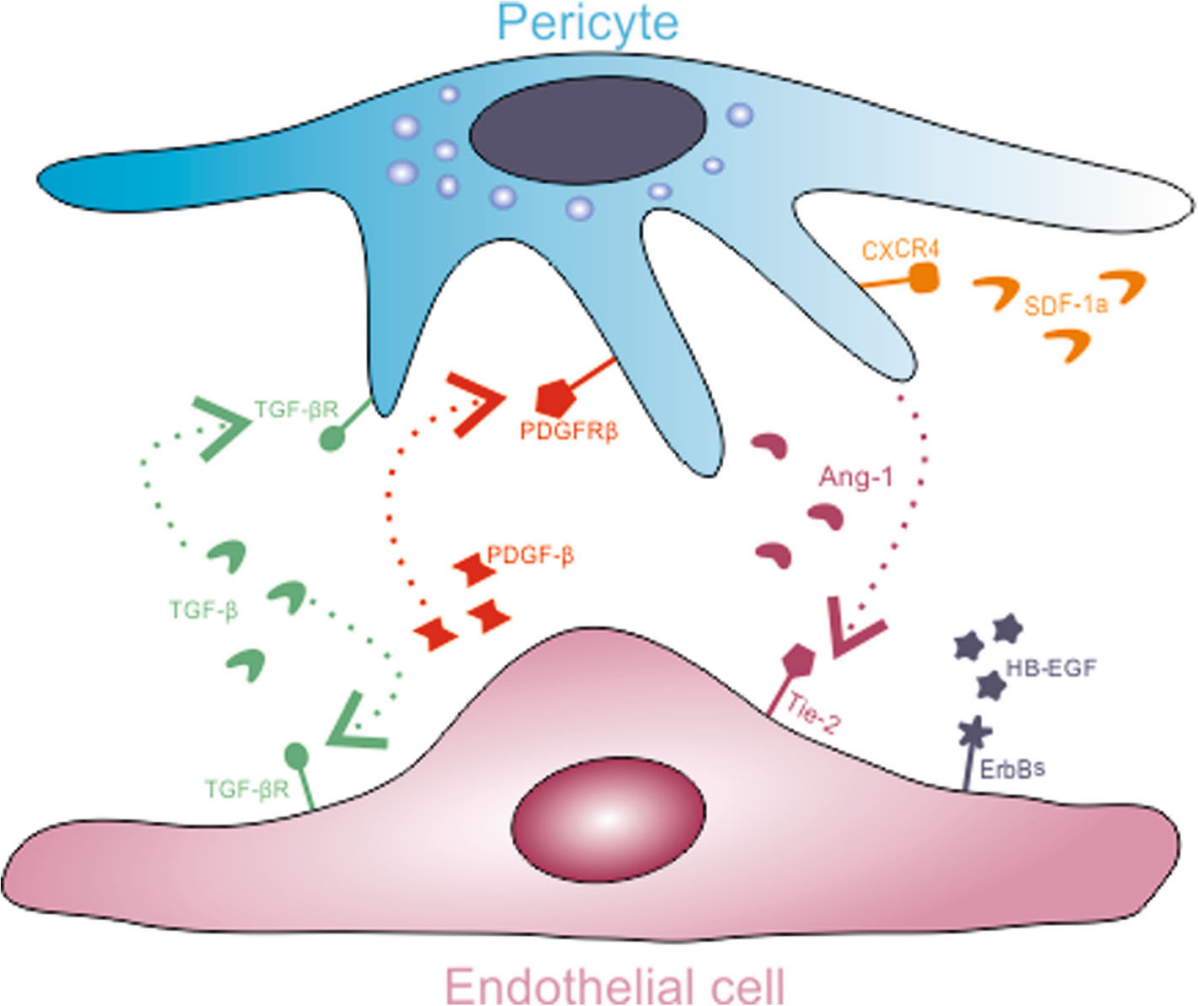
Signaling pathways between pericytes and ECs. The anatomical relationship and close interactions between pericytes and ECs are important for paracrine or juxtacrine signaling involved in processes of vascular development and stability. Recruitment of pericytes to the endothelium and their crosstalk with ECs is mediated by multiple pathways which are critically involved in embryonic and tumor angiogenesis. Pericyte recruitment to the endothelium is mediated by multiple ligand receptor complexes: PDGF-B/PDGFRb, SDF-1a/CXC4R, HB-EGF/ErbB, and Ang1/Tie-2. The cellular response to TGFb/TGFbR signaling axis is dependent on the composition of the receptor and the relative level of the ligand

Distribution of pericytes within the tissue microenvironment and their interaction with ECs are regulated by blood flow and oxygen content [26]. The anatomical relationship and close interactions between pericytes and ECs are important for paracrine or juxtacrine signaling involved in processes of vascular development and stability. Recruitment of pericytes to the endothelium and their crosstalk with ECs is mediated by multiple pathways which are critically involved in embryonic and tumor angiogenesis (Fig. 2).

During embryonic development, PDGF-B is released from ECs and binds to PDGFRb, expressed on the surface of developing pericytes. As a result, PDGFRb-positive pericytes (or their progenitors) are co-recruited to the angiogenic sprouts [5]. Postnatally, PDGF-B/PDGFRb signaling is involved in the pericyte recruitment to the tumors and in mural cell recruitment and vessel maturation in thalidomide-treated patients with hereditary hemorrhagic telangiectasia [5, 27].

Reciprocal orientation to the PDGF-B/PDGFRb signaling has an Angiopetin-1 (Ang-1)/Tie-2 paracrine loop where Ang-1 is produced by pericytes while Tie-2 is expressed on ECs. Ang-1 through the binding to the Tie-2 regulates maturation and integrity of ECs [28–30]. Pericyte-derived Ang-1 provides stabilizing signals to the endothelium that reduce vascular permeability and increase higher-order structure, quiescence, and functionality of the vessel network during development of the cardiovascular system and after vascular injury [5]. Additionally, Ang-1 may promote pericyte recruitment through heparin-binding epidermal growth factor (HB-EGF) signaling. HB-EGF, expressed by ECs binds to the EGF receptors (ErbBs) 1 and 4, expressed on pericytes and regulates their migration [31].

Another pathway recently implicated in pericyte recruitment is stromal-derived factor 1-a (SDF-1a)/CXCR4 axis which activation promotes pericyte migration in vitro and in vivo*,* during tumor angiogenesis [32]. SDF-1a acts synergistically with stem cell factor and interleukin (IL)-3 to mediate pericytes recruitment during the formation and maturation of endothelial tube [33] and activation of this molecular pathway can be stimulated by PDGF-B [32].

A huge number of studies have demonstrated the pivotal role of transforming growth factor beta (TGF-β) signaling for regulation of pericytes and ECs proliferation and differentiation as well as generation of new blood vessels [5, 25, 34–37]. TGF-β signaling is involved in pericytes: ECs crosstalk during vascular development in embryogenesis and carcinogenesis [5]. Both pericytes and ECs express receptors for TGF-β and are able to produce latent forms of this growth factor, but its activation is a consequence of the interplay between these cells [38]. Moreover, TGF-β, in cooperation with Notch, a well-known modulator of angiogenic sprouting [39, 40], regulate expression of N-cadherin in adhesion plaques between ECs and pericytes and control maturation of blood vessels [41]. A critical Notch ligand in this context is Jagged-1 (Jag-1) expressed on ECs and induced in pericytes as part of an autoregulatory loop of Jag-1/Notch3 signaling [42].

## Physiological function of pericytes

Interaction between pericytes and ECs is crucially important for the integrity and maintenance of the basement membrane of the vessel wall [43]. The contact between pericytes and ECs allows pericytes to regulate blood flow within vessels [44]. Pericyte to ECs ratio differs from tissue to tissue and is mainly dependent on blood pressure levels [45]. In the retina and central nervous system this ratio is 1:1, in the skin and lung the ratio is 1:10, while in the striated muscle this ratio is 1:100 [45]. It was recently revealed that microRNAs (miRNAs), are critical mediators in modulating perycite:EC crosstalk. MiR-145, identified in brain and kidney pericytes, targets transcription factor Friend leukemia integration 1 (Fli1) in ECs end regulates cell migration [46].

Additionally, pericytes mechanically regulate vessel wall integrity and serve as signaling mediators of ECs behavior. Pericytes in paracrine manner affect proliferation and maturation of ECs and are able to promote generation of new vessel sprouts when it is appropriate or to inhibit aberrant pro-angiogenic behavior of ECs when vessel sprouting is not required [30].

Pericytes:ECs interactions are affected not only by biochemical factors such as ligand–receptor kinetics, but also through the pericytes’ exertion of mechanical forces that are communicated to nearby ECs through either direct strain or indirect mechanical stiffening of the underlying nonlinear elastic substrata [47]. There is accumulating evidence that mechanical microenvironments, such as blood pressure, fluid shear stress, and cyclic strain, affect pericyte:ECs cross-talk and function.

When mechanical strain applied to ECs, ECs are activated and re-enter the cell cycle. Pericyte contraction, resulting with the attendant strain of the ECs environment, serves as an early mechanical cue that promotes activation of ECs and development of capillary-like endothelial sprouting [48].

Fluid shear stress induces up-regulation of “a disintegrin and metalloproteinase with thrombospondin motif 1 (ADAMTS-1)” in ECs, a metalloproteinase that is crucially important for vascular regression [49]. Pericytes, exposed to shear stress, provide adaptive and protective mechanism to endothelial damage by increasing expression of “tissue inhibitor of matrix metalloproteinase 3 (TIMP-3)”, which counter-regulates endothelial ADAMTS-1 and prevents matrix degradation enabling maintenance of vascular stability [50]. Interaction between pericytes and ECs under shear stress is regulated by miR-27. Flow-induced overexpression of miR-27 increased pericyte adhesion to ECs while inhibition of miR-27 reduced pericyte coverage of ECs tubes in vitro and pericytes coverage of endometrial vessels in the murine uterus in vivo [51].

Pericytes play an important role in intussusceptive angiogenesis, as well [52]. By electronic microscopy we observed that pericytes are recruited during the initial and final phases of vascular pillar formation in several organs [53, 54]. Similar as in sprouting angiogenesis, PDGF-B/PDGFRb and Ang-1/Tie-2 pathways were responsible for pericyte recruitment during intussusceptive angiogenesis [55]. Accordingly, we postulate that the recruitment of pericytes contributes either to the synthesis and mechanical stabilization of the transcapillary pillar core or to the maintenance of a low vascular permeability during intussusception [52].

Recently, it has been found that pericytes may regulate the diffusion of cells and proteins from the vessel to the surrounding tissue, influencing the infiltration of the immune cells in inflamed tissues [56–58]. Initially, pericytes were believed to be involved only in vasoconstriction. However, in the last fifty years, different functions in physiological processes are assigned to pericytes. First of all, pericytes co-create the walls of the microvessels (capillaries, terminal arterioles and postcapillary venules) since their long processes encircle the abluminal surface of these vessels and attribute structural integrity to the vessel wall [59]. Additionally, pericytes are involved in the preservation of vascular homeostasis, including regulation of blood flow, angiogenesis, structural stabilization of the vasculature, and vascular permeability. This is of particular importance in the central nervous system and retina, where pericytes form blood-brain barrier and blood-retinal barrier where pericyte density form a filter which protects brain and retina cells from potentially toxic blood-derived factors [45]. Many pericyte functions are tissue specific and depend on the morphological and functional characteristics of pericytes residing in specific microenvironments: pericytes regulate ultrafiltration in the kidneys [60], maintain the homeostasis of the vascular niche in bone marrow [61] and are involved in the remodeling of the extracellular matrix [ECM] in the fenestrated endothelium of the liver [62].

Moreover, emerging evidence demonstrates the involvement of pericytes in modulation of immune response [2]. Pericytes constitutively expressed major histocompatibility complex [MHC] class I but not MHC class II or the co-stimulatory molecules CD80 or CD86. However, in vitro IFN-γ treatment can induce the expression of MHC class II by all types of pericytes indicating their potential to act as professional antigen presenting cells. These findings are of particular importance since in vivo, in the presence of inflammatory cytokines [IFN-γ, IL-17, TNF-α and IL-1β] pericytes secrete a plethora of chemokines and cytokines and attract immune cells to the site of inflammation [2]. CD4 + IFN-γ producing Th1 cells, CD8+ cytotoxic T lymphocytes and natural killer (NK) cells migrate in the inflammatory microenvironment attracted by CXCL10 released by pericytes, while pericyte-derived CXCL8 and CXCL1 are involved in recruitment of neutrophils. Additionally, pericytes produce CCL2, CCL3 and CCL5 which bind to CCR2, CCR1 and CCR5 and modulate monocyte, macrophage, CD4 + Th1, CD8, and NK cell trafficking [63, 64]. Pericytes, activated by inflammatory cytokines, overexpress adhesion molecules that facilitate transendothelial migration of immune cells [65]. Pericytes promote “abluminal crawling” of neutrophils through the interaction between pericyte-expressed intercellular adhesion molecule 1 [ICAM-1] with neutrophil-expressed lymphocyte function-associated antigen 1 (LFA-1) [56]. At the same time, pericytes relaxation facilitates neutrophil extravasation [57]. Similarly, pericytes are implicated in modulating trafficking of T cells, as well. They control mature T cell transmigration across the endothelium from the thymus into circulation [2]. Lipopolysaccharide (LPS)-activated human brain pericytes significantly increase expression of both ICAM-1 and vascular cell adhesion molecule 1 (VCAM-1) which promote adhesion of peripheral blood lymphocytes to pericytes enabling their crosstalk. IFN-γ-stimulated human placental pericytes express program death ligand (PD-L1) and PD-L2 that binds to PD on activated T cells and suppresses their proliferation or renders them anergic in contact-dependent manner. Additionally, retinal pericytes may inhibit T cell proliferation and activation in paracrine manner, through the production of immunosuppressive IL-10 [66]. Importantly, in TGF-β dependent manner, pericytes are able to promote generation of immunomodulatory CD4 + CD25 + FoxP3+ regulatory T cells confirming their immunosuppressive potential [67].

A fact that pericytes are cells with immunomodulatory characteristics and that most of them are derived from mesoderm, indicated their resemblance with perivascular mesenchymal stem cells (MSCs), multipotent, self-renewable cells that, upon injury, promote tissue repair by suppressing immune response and inflammation [68–72]. The use of molecular markers to separate MSCs from other perivascular populations and evidences from refined lineage tracing models indicate that the perivascular MSC might originate pericytes in some organs. It was hypothesized that, upon tissue injury, pericytes, in a similar manner as MSCs, become activated in response to released inflammatory mediators and act as genuine MSCs to attenuate inflammation and promote tissue repair [73]. In line with this hypothesis is data obtained after the analysis of human bone marrow-derived MSC and human retinal CD146+ pericytes [23] that indicate similarities in morphology and differentiation potential between MSCs and pericytes. These two cell populations have the same morphology, share the expression of 22 cell surface molecules, and have the same capacity to differentiate toward osteogenic, adipogenic, and chondrogenic lineages in vitro [23]. However, perivascular MSCs and pericytes significantly differ in the expression of structural proteins: desmin and α-SMA. While capillary pericytes are consistently positive for desmin [both under culture conditions and in vivo], MSCs isolated from mouse, rat and human tissues are all negative for desmin [74–78]. The observation that only some subsets of pericytes express α-SMA, leads several researchers to postulate that α-SMA(+) pericytes are more likely to carry out a structural support at the blood vessel wall while α-SMA(−) pericytes possess MSC-like regenerative and immunomodulatory characteristics [23]. In line with these findings, further studies should be conducted paving the way for clarifying relationship between perivascular MSCs and pericytes, two immunosuppressive types of cells crucially involved in tissue repair and regeneration.

## Pathological roles of pericytes

Since pericytes regulate angiogenesis and inflammation, they play an important role in the pathogenesis of fibrosis, ischemic organ failure, tumor growth and metastasis, and their number was significantly altered in several pathologies including diabetes and diabetes-related complications, pulmonary hypertension and Alzheimer disease.

Pericytes may contribute to the development of fibrosis in two ways: directly by producing collagen [79] or indirectly by differentiating into myofibroblasts, α-SMA expressing and collagen producing cells, responsible for the expansion of fibrotic tissue [80]. Accumulating evidence indicates that PDGFRb and α-SMA-expressing pericytes may constitute a source of myofibroblast progenitors in the liver, kidney and the skin [81–84], indicating pericytes as myofibroblasts-generating cells. MiR-132 was recently identified as an important mediator involved in proliferation of pericyte-derived miofibroblasts and development of renal fibrosis. Analysis of fibrotic kidneys revealed significantly higher expression of miR-132 in pericyte-derived myofibroblasts while silencing of miR-132 counteracts the progression of renal fibrosis by selectively decreasing myofibroblast proliferation [85].

Pericytes are recruited into tumor blood vessels in PDGF-B/PDGFRb, HB-EGF/Erb and SDF-1a/CXCR4 dependent manner [27, 86, 87]. Being a ubiquitous part of the tumor microenvironment, pericytes regulate both neo-angiogenesis and vessel permeability, playing an important role in vascularization and blood supply of rapidly growing tumors [88]. In contrast to the healthy tissue vasculature, tumor vessels are characterized by irregular shape and a disorganized architecture with highly dysfunctional and leaky endothelial cell layers [89]. Also, the extent of pericyte coverage on tumor vessels is significantly reduced when compared to normal tissues. Moreover, pericytes are very firmly associated to the endothelial cells within tumors. It was assumed that hypoxia-induced expression of VEGF-A activates pericytes resulting with their consequent detachment from basement membrane of tumor vessels. In line with this hypothesis are data obtained after pharmacological blockade of VEGF-A showing increased pericyte coverage and attachment to the vascular wall, normalization of tumor vasculature [90] and an improved oxygen, drug, and nutrient delivery to the tumor [91].

Additionally, pericyte deficiency increases interstitial fluid pressure [5], which might promote metastasis enabling passive flow of detached tumor cells into the circulation through leaky endothelial cell layers. Accordingly, it was hypothesized that pericytes play an important role in suppression of metastasis being a part of a physical barrier to vascular dissemination and/or extravasation of malignant cells. Based on these findings, it can be concluded that modulation of pericyte number and activity could be considered as new approach in anti-tumor therapy since normalization of tumor vessel significantly improves drug and chemotherapy delivery to the tumor and prevents metastasis.

One of the earliest hallmarks of diabetes and related complications (limb ischemia, retinopathy, nephropathy, neuropathy, erectile dysfunction) is the loss of pericytes which causes regression of the microvasculature leading to leaking of fluids, leukocyte adhesion to the vasculature and hypoxia in the damaged area. Reduced number of pericytes seen in diabetic patients is a consequence of hyperglycemia-induced apoptosis of pericytes which mainly happens due to an increase in production of reactive oxygen species (ROS) that disrupt mitochondrial membrane of pericytes, resulting with the release of cytochrome C and the activation of the caspase-3 dependent apoptotic cascade. Another mechanism by which hyperglycemia-induced oxidative stress can cause pericyte loss is through the generation of advanced glycation end-products (AGEs). Through the binding to their receptors, expressed on the membrane of pericytes, AGEs induce apoptosis of pericytes, further contributing with their reduced number in diabetic patients [92].

The dysfunction, degeneration and loss of pericytes in diabetic patients are crucially important for the development of peripheral vascular complications, including diabetic limb ischemia. Mi-RNAs are considered as critical regulators of gene expression in this condition. Hyperglycemia and ischemia induce increased expression of miR-503 in ECs which is, within extracellular vesicles and microparticals, distributed to the pericytes. The integrin-mediated uptake of miR-503 in the recipient pericytes reduced expression of EfrinB2 and VEGF-A, resulting with the impaired migration and proliferation of pericytes and consequent progression of diabetic limb ischemia [93].

Diabetic retinopathy, a leading cause of blindness, is one of the most common complications of diabetes mellitus [94]. During the progression of diabetic retinopathy, due to the apoptosis of pericytes or due to their detachment from the basement membrane, pericyte to EC ratio reduces four times causing a significant decrease in the number of functional blood vessels as well as disruption of the blood retinal barrier. These changes lead to the development of non-proliferative phase of diabetic retinopathy as a result of an increase capillary leakage, macular edema and vessel occlusion. Proliferative diabetic retinopathy, characterized by the growth of abnormal new vessels from the retina to the posterior surface of the vitreous or the iris, develops secondary to capillary occlusion and represents one of the most usually causes of the blindness.

In similar manner as in diabetic retinopathy, hyperglycemia-induced alteration in number and function of renal pericytes results with the development of diabetic nephropathy, manifested by proteinuria, progressive decline in the glomerular filtration rate and increased arterial blood pressure. High glucose causes migration of peritubular pericytes away from the capillary into the interstitial space where they differentiate into collagen-producing myofibroblasts significantly contributing to the development of tubulointerstitial fibrosis and renal dysfunction. [59] It is assumed that hyperglycemia-induced increased generation of ROS and enhanced expression of AGEs up-regulate expression of major pro-fibrogenic cytokine TGF-β in peritubular pericytes, podocytes and mesangial cells [95, 96]. This is followed with the increased production and accumulation of collagen and fibronectin with the consequent development of tubulointerstitial fibrosis. Lumen of the peritubular capillaries and glomerulus become occluded leading to an increase in blood pressure, while expansion of mesangial cell matrix may result in the development of glomerular scarring significantly reducing the surface area for glomerular filtration.

Prolonged and incomplete wound healing, caused by dysfunction of pericytes and impaired angiogenesis, has been observed as a complication of diabetes mellitus [97]. As a non-invasive method, negative pressure wound therapy (NPWT) has been demonstrated to accelerate wound healing by promoting angiogenesis [98]. NPWT induced overexpression of angiogenin-1 (Ang-1), Tie-2, α-SMA and collagen type IV, and significantly increased blood flow perfusion and microvessel maturation. Ang-1, primarily expressed in pericytes, serves as a vascular pro-maturation factor which binds to Tie-2 and regulates pericyte recruitment to endothelium tubes [99]. In Ang-1/Tie-2 dependent manner, NPWT induced proliferation of α-SMA + pericytes and promoted assembly of basement membrane enabling formation of an enveloped endothelium tube [98]. Increased number of pericytes was accompanied with enhanced deposition of collagen type IV, major basement membrane component that stabilizes vascular structure and regulates vessel morphogenesis [100]. Pericytes and collagen type IV complemented each other and promoted the maturation of new blood vessels resulting with an increased blood flow perfusion and accelerated healing of diabetic wounds [98].

Hyperglycemia-induced apoptosis of pericytes contributes to the development of neuropathy and erectile dysfunction in diabetic patients. Reduced number of pericytes leads to the disorganization of ECs, resulting in decreased perfusion of peripheral nerves and consequent hypoxia. The hypoxia is, in turn, responsible for oxidative stress and apoptotic cell death of pericytes which results with microangiopathies of the endoneurial capillaries [59]. Similarly, decrease in penile pericyte number, associated with an increase in corpus cavernosum sinusoidal permeability and erectile dysfunction, were observed in animal models of diabetes mellitus type 1 [101] confirming important role of pericytes in the development of diabetes-related vascular pathobiology.

Pericytes have an important role in the etiology of ischemic organ failure [59]. In the ischemic kidneys, pericytes differentiate into collagen producing myofibroblasts contributing to the scar formation and the development of the interstitial renal fibrosis [102]. Similarly, pericytes are recruited to the scar tissue of infarcted myocardium and are involved in cardiac tissue remodeling [59]. In cerebral ischemia, pericytes produce matrix metalloproteinase (MMP)-9 resulting with the breakdown of the blood brain barrier [103]. Dysfunction of pericytes is also observed at the blood brain barrier in patients with Alzheimer’s disease [104]. Ischemia-induced apoptosis of pericytes leads to neurodegeneration, caused by decreased cerebral blood flow and vascular breakdown accompanied with an increased accumulation of damaging molecules in the brain [105].

Pericytes have an important pathological role in the development of pulmonary hypertension. When compared to the vessels of healthy controls, an increased number of pericytes is found in pulmonary arteries of patients suffering from pulmonary hypertension. It was assumed that these additional pericytes serve as a source of smooth muscle-like cells leading to the excessive remodeling of the pulmonary vasculature, endothelial dysfunction and development of PAH [106].

## Potential of pericyte-based therapy: What have we learned from animal models?

Strong evidence for pericytes’ participation in physiological, as well as in pathological conditions reveals a broad potential for the therapeutic use of these cells [107, 108]. Several animal studies indicate that pericytes can contribute positively to healing in animal models of muscle damage, myocardial infarction, diabetic retinopathy, bone and skin injury [13, 109, 110].

Pericyte-based therapy holds great potential to enhance the healing of injured muscles. Human pericytes (CD146+, NG2+ and PDGFR+) were able to produce myofibres contributing to the muscle regeneration after transplantation in mice with cardiotoxin-induced muscle damage [13]. Similarly, human pluripotent stem cells (hPSC)-derived pericytes successfully engrafted into the vasculature and the muscle tissue of the recovering limb and promoted vascularization and muscle regeneration [110].

Although pro-fibrotic role of pericytes were well documented in animal models of chronic kidney injury and diabetic nephropathy [101, 102], pericyte application to a mouse model of myocardial infarction significantly reduced fibrosis, decreased infiltration of inflammatory cells and increased angiogenesis and blood supply to the ischemic heart which resulted in improved recovery and contractility of damaged myocardium [58]. It seems that in this model the microenvironment of an ischemic heart promotes immunomodulatory and pro-angiogenic characteristics of pericytes and hinders their differentiation into myofibroblasts, confirming that pericyte function and heterogeneity are heavily influenced by the surrounding tissue environment [109].

Transplantation of human adipose tissue-derived pericytes (α-SMA+, PDGFR+NG2+) protects against diabetic retinopathy in a mouse model by promoting angiogenesis and vascular support [111]. This was mainly a consequence of the pericyte replacement in retinal blood vessels since loss of pericytes was the main reason for the collapse of the vasculature and developed retinopathy. Despite of these promising results, data obtained in RETICELL clinical trial showed that intravitreal transplantation of human MSCs, which act as pericytes in pathologic conditions of the eye, managed to restore ocular function in patients with retinitis pigmentosa up to 3 months after injection, but this improvement has been lost with time [112].

Promising results of pericyte-based therapy have been observed in murine models of skin and bone injury, as well [113]. The application of human adipose-derived pericytes (α-SMA+, PDGFR+, NG2+ and Ang1+) on wounded skin of the rats had beneficial effects due to the increased angiogenesis, extensive collagen deposition and re-epithelialization [113]. Similarly, both mouse (CD146 + NG2 + CD45-) and human (CD146 + CD34-CD45-CD31-) adipose-derived pericytes enhanced bone healing and regeneration in mouse bone injury models [114, 115].

## Conclusions

The body of knowledge illustrates the importance of pericytes in the regulation of homeostatic and healing processes in the body. The functions and capabilities of pericytes are impressive and, as yet, incompletely understood. Molecular mechanisms responsible for pericyte-mediated regulation of vascular stability, angiogenesis and blood flow are well known while their regenerative and immunomodulatory characteristics are still not completely revealed. An aberrant number or function of pericytes is associated with the development of fibrosis, diabetes-related complications, ischemic organ failure, pulmonary hypertension, Alzheimer’s disease, tumor growth and metastasis. Recent in vivo studies show that the transplantation of pericytes can positively influence the healing of bone, muscle and skin and can support revascularization in a mouse model of diabetic retinopathy. However, the differences in their phenotype and function as well as the lack of standardized procedure for their isolation and characterization limit their use in clinical trials and until now pericytes were in regenerative medicine mainly used for repopulation of vascular grafts [116]. Critical to further progress in clinical application of pericytes will be identification of tissue specific pericyte phenotype and function, validation and standardization of the procedure for their isolation that will enable establishment of precise clinical settings in which pericyte-based therapy will be efficiently applied. The first steps in this direction have recently been made. A new, clinical-grade method for isolation of NG2 expressing human pericytes from cadaveric kidneys has recently been described. Human kidney-derived pericytes, through the production of hepatocyte growth factor, induced accelerated repair in a tubular epithelial wound scratch assay in vitro and gave protection against the development of acute kidney injury in vivo [117]. It can be expected that, based on these promising results, new protocols for isolation of tissue-specific pericytes will be established that will enable clinical application of pericytes in cell based therapy.

## Abbreviations

AGEs: advanced glycation end-products
ECs: endothelial cells
Gli1: glioma-associated oncogene
hPSC: human pluripotent stem cells
LFA-1: lymphocyte function-associated antigen 1
MMP: matrix metalloproteinase
MSCs: mesenchymal stem cells
NG2: nerve-glial antigen-2
NK: natural killer
PDGFRβ: platelet-derived growth factor receptor β
PD-L1: program death ligand
RGS5: the regulator of G-protein signaling-5
ROS: reactive oxygen species
Tbx18: T-box transcription factor TBX18
TGF-β: transforming growth factor beta
VCAM-1: vascular cell adhesion molecule 1
vSMCs: vascular smooth muscle cells
α-SMA: α-smooth muscle actin
